# Efficacy of 6‐mm diameter fully covered self‐expandable metallic stents in preoperative biliary drainage for pancreatic ductal adenocarcinoma

**DOI:** 10.1002/deo2.55

**Published:** 2021-09-08

**Authors:** Fumiya Kataoka, Dai Inoue, Masato Watanabe, Keita Fukuda, Tsubasa Nobusawa, Kayo Umemura, Natsuki Miura, Takuya Yokota, Astushi Yoshioka, Kohei Shimoji, Ayano Nakazono, Hideyuki Horike, Yuki Ogura, Tatsuya Hayashi, Yasuhiro Morita, Shin Namiki

**Affiliations:** ^1^ Department of Gastroenterology Tokyo Metropolitan Tama Medical Center Tokyo Japan; ^2^ Department of Digestive Surgery Tokyo Metropolitan Tama Medical Center Tokyo Japan

**Keywords:** malignant biliary obstruction, pancreatic ductal adenocarcinoma, preoperative biliary drainage, recurrent biliary obstruction, self‐expandable metallic stent

## Abstract

**Objectives:**

Plastic stents (PS) used for preoperative biliary drainage (PBD) of pancreatic ductal adenocarcinomas (PDAC) tend to be associated with a high incidence of recurrent biliary obstruction (RBO). Although 10‐mm diameter fully covered self‐expanding metallic stents (FCSEMS) have come into use, vigilance is still required to prevent complications, such as cholecystitis and surgical site infection. The present study examined the efficacy and safety of the 6‐mm diameter FCSEMS for PBD.

**Methods:**

The present retrospective study compared the incidence of complications associated with the use of 6‐mm FCSEMS and PS. The inclusion criteria were a diagnosis of PDAC and preoperative endoscopic biliary tract drainage performed at our institution between April 2012 and June 2019.

**Results:**

Of the 51 patients enrolled, 25 and 26 patients received a PS and a 6‐mm FCSEMS, respectively. The RBO incidence was significantly lower in the 6‐mm FCSEMS group (7.7%) than in the PS group (40.0%) (*p* = 0.009), and time to RBO was significantly longer in the 6‐mm FCSEMS group (HR = 6.008, *p* = 0.021). The patency rate at three months after stent placement was significantly higher in the latter group (83.5% vs. 45.3%, *p* = 0.009, Log‐rank test). The groups did not differ significantly in terms of complications associated with PBD, such as cholecystitis and surgical site infection.

**Conclusion:**

The present findings suggested that the 6‐mm FCSEMS may be an effective drainage device for use in PBD in PDAC treatment.

## INTRODUCTION

Pancreatic ductal adenocarcinoma (PDAC) is the fourth most common cause of cancer‐related mortality in Japan. The only curative treatment option is surgery. Patients with PDAC, especially in the pancreatic head, frequently have obstructive jaundice. The pros and cons of preoperative biliary drainage (PBD) for patients with obstructive jaundice are still debated. Several reports have pointed out the need for caution when carrying out a PBD routinely in patients with PDAC with obstructive jaundice because it might increase the risk of complications such as cholecystitis and surgical site infection (SSI).^1^ ^‐–^ ^4^ For these reasons, some guidelines on pancreatic cancer treatment recommend performing early surgery without PBD, reserving the procedure only for patients with severe jaundice, cholangitis, or planned neoadjuvant chemotherapy (NAC).^5^, ^6^

Severe jaundice can cause blood coagulation abnormalities and raise the risk of perioperative bleeding.^1^, ^7^ However, recent studies reported some convincing evidence that NAC can improve the prognosis of patients with borderline resectable PDAC.^8^ Moreover, a Japanese group reported that NAC improved overall survival in patients with PDAC for whom upfront curative surgery was considered appropriate.^9^

When performing a PBD, it is important to choose a type of biliary stent with a low risk of endoscopic retrograde cholangiopancreatography (ERCP)‐related complications, such as cholangitis, migration, cholecystitis, and post‐endoscopic retrograde cholangiopancreatography pancreatitis (PEP) and postoperative complications, such as SSI.

At our institution, plastic stents (PS) were previously used for PBD in patients with PDAC. These have been replaced now with the newly released, smaller, 6‐mm diameter fully‐covered self‐expanding metallic stents (FCSEMS) aimed at reducing stent‐related complications. The present study examined the efficacy of the 6‐mm FCSEMS for PBD in patients with PDAC.

## METHOD

The present retrospective study compared the efficacy and safety of the 6‐mm FCSEMS and PS. The 6‐mm FCSEMS used in this study was HANAROSTENET® Biliary (Microvasive Endoscopy; Boston Scientific, Natick, Massachusetts, USA). Patients with a diagnosis of PDAC, who underwent PBD for distal malignant biliary obstruction at Tokyo Metropolitan Tama Medical Center between June 2012 and March 2020, were collected. Of these patients, those satisfying the following criteria were enrolled: histologically confirmed PDAC, no treatment for PDAC at the time of biliary tract drainage, localized tumor without distant metastasis, and ability to tolerate curative surgery. The exclusion criteria were a failure of endoscopic biliary stent placement due to technical difficulties, use of a stent other than a PS or 6‐mm FCSEMS, and unsuitability for curative surgery.

The Institutional Review Board of Tokyo Metropolitan Tama Medical Center approved this study and waived the requirement for written informed consent because of its retrospective, non‐interventional design. The patients were given the opportunity to opt‐out via our hospital website.

### Evaluation of outcomes

Clinicopathological data were collected from the medical records. The variables examined in this study were age, gender, body mass index, total bilirubin level before biliary tract drainage, localization of the primary pancreatic tumor, pathological stage according to the TNM classification (7th edition), date of biliary tract drainage, type of biliary stent, and surgical findings, such as the date of surgery, surgical method, operative time, surgical bleeding, the incidence of postoperative complications, and pathological findings of the tumor.

To evaluate the efficacy of the stents, recurrent biliary obstruction (RBO) and time to RBO (TRBO) were evaluated as primary endpoints and compared between the PS and 6‐mm FCSEMS groups. RBO was defined as a composite endpoint of either occlusion or migration, and TRBO was defined as the time from FCSEMS/PS placement to biliary obstruction recurrence.

To evaluate the safety of the biliary stents, the incidence of complications other than RBO, such as cholecystitis, non‐occlusive cholangitis, and pancreatitis after stent placement, were also investigated. Furthermore, to evaluate the impact on surgery of the different stent models, the R0 resection rate, operative time, surgical bleeding, and incidence of postoperative complications were analyzed.

### Statistical analysis

Categorical data were analyzed using the chi‐square test or Fisher's exact test while continuous variables were compared using the Mann–Whitney *U*‐test. TRBO was estimated using Kaplan–Meier analysis with the log‐rank test. All statistical analyses were conducted using SPSS Statistics 23.

## RESULTS

**FIGURE 1:**
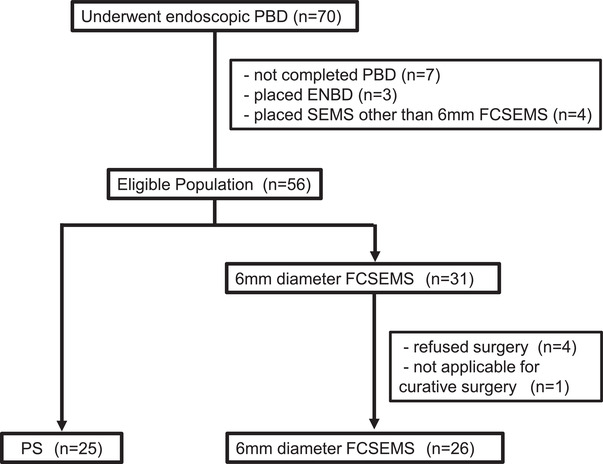
Patient enrollment and group allocation PS, plastic stent; SEMS; self‐expandable metal stent; RBO, recurrent biliary obstruction; ENBD, Endoscopic nasobiliary drainage; and ERBD, Endoscopic retrograde biliary drainage

Between April 2012 and March 2020, 70 patients received the diagnosis of PDAC and underwent endoscopic PBD. Of these, seven did not complete the procedure due to technical difficulties, and seven were excluded because a stent other than a PS or 6‐mm FCSEMS was used (three patients underwent endoscopic nasobiliary drainage and four patients received another type of SEMS), four patients declined to receive chemotherapy, and one patient was judged to be unsuitable for curative surgery. Fifty‐one patients met the selection criteria; of these, 25 received a PS, and 26 received a 6‐mm FCSEMS (Figure 1). In the PS group, 7Fr FleximaTM Biliary Stent System (Boston Scientific) was used in 22 patients, 8.5Fr FleximaTM Biliary Stent System (Boston Scientific) was used in one patient, and a double pig‐tail type bile duct stent was used in two patients.

Table 1 shows the patient characteristics. No significant difference was seen in the age, primary tumor diameter, body mass index, or total bilirubin level. All the patients received a PS between 2012 and 2016. Since 2017, the 6‐mm FCSEMS has largely replaced the PS at our hospital, as mentioned previously. The groups did not differ significantly in terms of the clinical stage according to the TNM classification (7th edition). However, according to the National Comprehensive Cancer Network criteria of resectability at diagnosis, significantly more patients in the 6‐mm FCSEMS group were classified as having a resectable tumor. The median tumor diameter also tended to be smaller in the 6‐mm FCSEMS group. These differences were apparently attributable to improvements in the diagnostic imaging technology of computed tomography and endoscopic ultrasonography since 2017 allowing small, early‐stage PDAC to be detected precisely.

**TABLE 1:** Patient characteristics

		**PS (*n* = 25)**	**6 mm FCSEMS (*n* =26)**	***p*‐value**
Gender (male/female)		13/12	12/14	0.782
Age, years, mean (range)		70.0 (45–83)	76.5 (42–86)	0.079
Primary tumor diameter, median (range)		3.7 (1.5–6.0)	3.0 (2.0–5.5)	0.098
Body mass index, median (range)		21.0 (15.6–26.8)	22.2 (17.4–34.2)	0.134
Initial total bilirubin (mg/dl), median (range)		8.4 (0.4–16.0)	8.9 (0.6–27.8)	0.799
Pathological TNM stage (%)				
	T1	1 (4)	0	0.490
	T2	14 (56)	21 (80.8)	0.075
	T3	9 (36)	5 (19.2)	0.220
	T4	1 (4)	0	0.490
	N0	5 (20)	9 (34.6)	0.349
	N1	11 (44)	8 (30.8)	0.393
	N2	10 (36)	8 (30.8)	0.771
Operative method (%)				
	PD/SSPPD	24 (96)	25 (96.2)	1.000
	PpPD	1 (4)	1 (3.8)	1.000
year of PBD (%)				
	2012–2016	23 (92)	0	0.000*
	2017–2020	2 (8)	26	0.000*
Staging (%)				
	R	15 (60)	23 (88.5)	0.027*
	BR	10 (40)	2 (7.6)	0.009*
	LA	0	1 (3.8)	1.000
Clinical success rate		100	100	
Bile duct diameter		16 (11–21)	14 (12–22)	0.21
Stent length (%)				
	5 cm	11 (45.8)	‐	
	6 cm	‐	15 (57.7)	
	7 cm	13 (54.2)	‐	
	8 cm	‐	11 (42.3)	
Tumor encasement of the cystic duct		3 (12)	2(7.7)	0.668
EST		10 (40)	25 (96.2)	<0.001
Average medical cost/patient (Yen)		700,000	705,000	

Abbreviations: BR, borderline resectable; FCSEMS, fully covered self‐expandable metal stents, LA, locally advanced; PD, pancreatoduodenectomy; PpPD, pylorus‐preserving pancreatoduodenectomy; PS, plastic stent, RBO, recurrent biliary obstruction; TNM, TNM classification (7th edition); SSPPD, subtotal stomach‐preserving pancreatoduodenectomy.

* *p* < 0.05.

In terms of the surgical method used, a pancreatoduodenectomy (PD) was performed in 11 patients, subtotal stomach‐preserving PD (SSPPD) in 38 patients, and pylorus‐preserving PD (PPPD) in two patients. Bile duct diameter and tumor encasement of the cystic duct showed no difference between the two groups, and only the rate of endoscopic sphincterotomy was significantly higher in the 6‐mm FCSEMS group. A comparison of the medical cost showed that FCSEMS was more expensive to perform than PS, but that the rate of RBO was higher in the PS group, with the result that the overall cost was about the same for both groups.

**FIGURE 2:**
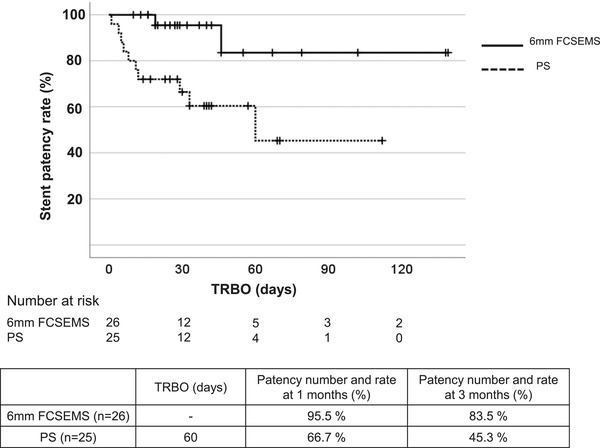
Stent patency rate in the plastic stents (PS) group and 6‐mm FCSEMS group SEMS: self‐expandable metal stent; RBO, recurrent biliary obstruction; TRBO, time to recurrent biliary obstruction

Table 2 and Figure 2 show the incidence of RBO and TRBO, the primary endpoints of the present study. The RBO incidence was significantly lower in the 6‐mm FCSEMS group (7.7%) than in the PS group (40.0%, *p* = 0.009; Table 2). While occlusion and migration occurred moderately in the PS group, occlusion was seen in only two patients (7.7%) and migration in none of the patients in the 6‐mm FCSEMS group despite the smaller diameter of the stent. Re‐intervention was performed in all the patients with RBO. As for the stents used at the time of re‐intervention, endoscopic nasobiliary drainage and endoscopic retrograde biliary drainage were used in five patients each in the PS group while the 10‐mm diameter FCSEMS was used in two patients in the 6‐mm FCSEMS group. TRBO was significantly longer in the 6‐mm FCSEMS group. The median TRBO was 60 days in the PS group while it did not reach the median value during the observation period in the 6‐mm FCSEMS group (HR: 6.008; 95% CI: 1.31–28.47; *p* = 0.021). The patency rate at three months from stent placement was also significantly higher in the 6‐mm FCSEMS group (83.5% vs. 45.3%; *p* = 0.009, log‐rank test) (Figure 2).

**TABLE 2:** The incidence of RBO

		**PS (*n* = 25)**	**6‐mm FCSEMS (*n* = 26)**	***p*‐value**
Incidence		10 (40)	2 (7.7)	0.009*
Cause	Occlusion	4 (16)	2 (7.7)	0.419
	Migration	6 (24)	0	0.010*
			
Onset	Early (<30 days)	9 (36)	1 (3.8)	0.005*
	Late (≥31 days)	1 (4)	1 (3.8)	1.000
Re‐intervention		10 (40)	2 (7.7)	0.009*
Content of Re intervention	ENBD	5 (20)	0	0.023*
	ERBD	5 (20)	0	0.023*
	10‐mm FCSEMS	0	2 (7.7)	0.490

Abbreviations: ENBD, endoscopic nasobiliary drainage; ERBD, endoscopic retrograde biliary drainage; PS, plastic stent; RBO, recurrent biliary obstruction; SEMS, fully covered self‐expandable metal stents.

* *p* < 0.05.

Table 3 shows the incidence of complications other than RBO in both groups. The severity grading of complications following stent placement followed the 2014 Tokyo Criteria.^10^ Complications other than RBO occurred within 30 days from stent placement. Although pancreatitis occurred in 15.4% of patients in the 6‐mm FCSEMS group, the severity was mild, and stent removal was not necessary in all the cases. A few, non‐occlusive cases of cholangitis were seen in both groups, but all the cases were mild to moderate and resolved with antibiotic therapy and a few days of fasting. Only one case of cholecystitis was seen in the PS group, and none were seen in the 6‐mm FCSEMS group.

**TABLE 3:** Adverse events other than RBO

		**PS (*n* = 25)**	**6‐mm FCSEMS (*n* = 26)**	***p*‐value**
Incidence (%)		3 (12)	6 (23.1)	0.465
Pancreatitis	Mild	1 (4)	4 (15.4)	0.350
	Moderate	0	0	‐
Non‐occlusion cholangitis	Mild	0	0	‐
	Moderate	1 (4)	2 (7.7)	1.000
Cholecystitis	Mild	0	0	‐
	Moderate	1 (4)	0	0.490

Abbreviations: FCSEMS, fully covered self‐expandable metal stents; RBO, recurrent biliary obstruction.

Table 4 shows the NAC and the factors related to surgery and postoperative complications in both groups. Six patients in the 6‐mm FCSEMS group, including one with a locally advanced tumor and five with borderline resectable tumors, received NAC (GEM+S‐1 was performed in five patients, and GEM+nab‐PTX was performed in one patient. No patient in the PS group received NAC). There was no significant difference in the analysis of the chemotherapy regimens, but there was a significant overall difference. The R0/R1 resection rate, operative time, and incidence of postoperative complications, such as SSI, did not differ significantly between the groups. However, the amount of surgical bleeding was significantly larger in the PS group, possibly due to the generally larger tumor size in this group.

**TABLE 4:** Neoadjuvant chemotherapy (NAC) and factors regarding surgery

		**PS (*n* = 25)**	**6‐mm FCSEMS (*n* = 26)**	***p*‐value**
Neoadjuvant chemotherapy				
Total		0	6 (23.1)	0.023*
GEM+S‐1		0	5 (19.2)	0.051
GEM+nab‐PTX		0	1 (3.8)	1.000
Resection rate (%)				
R0		17 (68)	21 (80.8)	0.349
R1		8 (32)	5 (19.2)	0.349
Surgery waiting time (days), median (range)		33.0 (16‐112)	38.5 (13‐138)	0.821
surgery time (minutes), median (range)		478 (342‐666)	516 (363‐782)	0.572
Surgical bleeding (ml), median (range)		1211 (291‐9588)	878 (373‐7577)	0.044*
Postoperative complications (%)				
	Total	10 (40)	11 (42.3)	1.000
	SSI	2 (8)	4 (15.4)	0.668
	Pancreatic fistula	2 (8)	3 (11.5)	1.000
	Chyiorrhea	5 (20)	4 (15.4)	0.726

Abbreviations: FCSEMS, fully covered self‐expandable metal stents; NS, not significant.

* *p* < 0.05.

## DISCUSSION

Because previous studies demonstrated that SEMS achieved a longer patency period and had a better safety profile than PS, they are now more commonly used for PBD.^11^, ^12^, ^13^, ^14^ While reports of the utility of SEMS are numerous, adverse events associated with their use have not been discussed despite their relatively high frequency. For example, the incidence of cholecystitis after SEMS placement is reportedly 6.9%–10.8% in patients with distal malignant biliary obstruction.^15^, ^16^, ^17^ Nakai et al. suggested that the axial force (AF) exerted by SEMS increases the risk of cholecystitis.^15^

PEP is also another important adverse event associated with SEMS placement. The frequency of pancreatitis after SEMS was reportedly 1.8%–8.3%,^18^ apparently due to the high AF after SEMS placement compressing the orifice of the pancreatic duct and increasing the incidence of pancreatitis.^15^, ^19^ These previous reports of adverse events associated with SEMS are based on clinical data on the use of 10‐mm diameter SEMS, and no studies thus far have reported the performance and safety of the 6‐mm FCSEMS.

The AF varies little regardless of the SEMS diameter. A previous study demonstrated that a high AF is a risk factor of PEP. Thus, it is unlikely that the PEP occurrence rate would increase with the use of the 6‐mm diameter FCSEMS, as shown by the absence of any significant difference in the PEP rate between groups.

Another previous study reported that tumor growth in the orifice of the cystic duct and a high AF are risk factors of post‐ERCP cholecystitis.^15^ The absence of post‐ERCP cholecystitis, differences in tumor involvement in the orifice of the cystic duct, and the AF suggested that the stent diameter was significantly related to the development of post‐ERCP cholecystitis.

Preventing ERCP and postoperative complications is important in PDAC surgery. A series of studies^20^, ^21^ found that the SSI rate increased after SEMS placement. In the present study, the SSI rate did not differ significantly between the PS and 6‐mm FCSEMS groups. Previous studies reported an SSI rate of 11%–31% and 5%–14% in their SEMS and PS groups, respectively.^14^, ^20^ ^22^, ^23^ In contrast to these findings, the rate of postoperative complications, including SSI, was not significantly higher in the 6‐mm FCSEMS group than in the 10‐mm SEMS group, suggesting that the 6‐mm FCSEMS is safe for use in PBD because it is associated with a relatively low incidence of procedural and surgical complications.

The importance of NAC for pancreatic cancer is being increasingly recognized, and for appropriate PBD, which is necessary for safe, preoperative chemotherapy, the choice of the stent is important. Sasahira et al. reported that stent‐related complications occurred in 30% of cases of PS use at approximately one month after placement.^24^ Togawa et al. also reported that the RBO rate at 4–8 weeks after PS placement was clearly worse than after SEMS placement.^25^ These previous reports demonstrated clearly that PS is unsuitable for use in PBD before NAC, leaving next to be answered, the question of whether the 6‐mm FCSEMS is suitable for use with NAC despite the smaller diameter. Although the present study demonstrated a better TRBO in the 6‐mm FCSEMS group, longer patency is required for NAC than in upfront surgery.

The results of the Prep‐02/JSAP05 study may provide a basis for determining the requisite duration of patency for NAC. This phase II/III trial of NAC with gemcitabine and S‐1 versus upfront surgery showed a significant survival benefit of NAC in patients with resectable PDAC. The patients received two cycles of chemotherapy (21 days per cycle) and underwent surgery two to 4 weeks after the last chemotherapy administration. Because the schedule includes the preoperative period, the duration of stent patency needs to be at least three months, especially in patients with resectable PDAC receiving NAC. In our study, the median duration of stent patency with 6‐mm FCSEMS was not able to be estimated due to the low RBO rate. In six patients with PDAC at our hospital who were excluded from the present study for not having received surgery, a 6‐mm FCSEMS was left in situ longer than in patients receiving a curative operation because the stent was not removed unless RBO occurred or the patients died. The median TRBO in these six patients was 162 days, and the patency duration ranged from 105 to 210 days. All the patients thus achieved more than 3 months of patency. Stent‐related complications can also delay NAC induction, possibly worsening the prognosis. For such reasons as these, the 6‐mm FCSEMS may be a suitable model for PBD in patients receiving NAC.

The choice of the biliary stent model may be influenced to some degree by cost. In their comparison of SEMS and PS, Timothy et al. concluded that there was no difference in cost and that SEMS may be a good choice given the increasing importance of NAC in the treatment of PDAC.^26^

The present study has several limitations. First, it was a retrospective study at a single institution, and the patient pool was relatively small. Second, the 6‐mm FCSEMS and 10‐mm SEMS were not compared directly. Research assessing the relative merits of these two models is warranted. Third, chemotherapy may have affected stent patency. For example, NAC may increase the risk of stent deviation due to tumor shrinkage or have an immunosuppressive effect and thereby increase the risk of retrograde cholangitis.

The present study was the first to demonstrate the efficacy and safety of the 6‐mm FCSEMS for use in PBD in PDAC treatment. The findings demonstrated that the RBO incidence associated with 6‐mm FCSEMS use was lower than that associated with PS use and may decrease cholecystitis and PEP occurrence after stent placement, a feature which is clearly lacking in conventional SEMS.

In conclusion, the 6‐mm FCSEMS was found to be an effective and safe drainage method in patients with PDAC before surgery.

## CONFLICTS OF INTEREST

The authors declare that they have no conflict of interest.

## FUNDING INFORMATION

None.
